# Weight change and 15 year mortality: results from the European Prospective Investigation into Cancer in Norfolk (EPIC-Norfolk) cohort study

**DOI:** 10.1007/s10654-017-0343-y

**Published:** 2017-12-20

**Authors:** Angela A. Mulligan, Marleen A. H. Lentjes, Robert N. Luben, Nicholas J. Wareham, Kay-Tee Khaw

**Affiliations:** 1 Strangeways Research Laboratory, European Prospective Investigation into Cancer and Nutrition, Department of Public Health and Primary Care, University of Cambridge, Worts Causeway, Cambridge, UK; 2 EPIC, Department of Gerontology, Addenbrooke’s Hospital, School of Clinical Medicine, University of Cambridge, Cambridge, UK; 3 MRC Epidemiology Unit, Cambridge, School of Clinical Medicine, Institute of Metabolic Science, University of Cambridge, Cambridge Biomedical Campus, Cambridge, UK

**Keywords:** Weight change, Weight loss, All-cause mortality, CVD mortality, Cancer mortality, EPIC-Norfolk

## Abstract

Studies have reported a higher mortality risk associated with weight loss, particularly in middle-aged and older adults, although some of these studies did find that gaining weight was also associated with an increased mortality risk. We examined changes in weight in relation to mortality in a prospective population-based cohort study of men and women, resident in Norfolk, UK. Participants were assessed at baseline (1993–1997) and at a second examination (1998–2000), as part of the European Prospective Investigation into Cancer and Nutrition (EPIC-Norfolk) study, and followed up to 2015 for mortality. Participants with a self-reported history of cancer or cardiovascular disease, body mass index < 18.5 kg/m^2^ or missing data on adjustment variables, at either time-point were excluded, leaving 12,580 participants, aged 39–78 in 1993–1997, eligible for analyses. Cox proportional hazards models were used to determine Hazard Ratios (HRs) for all-cause (2603 deaths), cardiovascular (749 deaths), cancer (981 deaths), respiratory (226 deaths) and other causes of mortality (647 deaths) by categories of weight change. After multivariate adjustment, the HRs (95% CIs) for all-cause mortality for men and women who lost more than 5 kg were 1.85 (1.48–2.31) and 1.64 (1.31–2.05) respectively. Higher hazards were also found for specific causes of mortality and weight loss > 5 kg. Similar associations were observed after excluding deaths in the first 5 years of follow-up. Results for weight gain were inconclusive. We conclude that objectively measured weight loss, but not weight gain, was associated with subsequent higher mortality risk in this population-based study of middle-aged and elderly men and women. However, undiagnosed, pre-existing disease and the inability to account for weight cycling need to be remembered when interpreting these results. Unravelling the causal pathways underlying this association will require more detailed studies, including that of changes in body composition.

**Electronic supplementary material:**

The online version of this article (10.1007/s10654-017-0343-y) contains supplementary material, which is available to authorized users.

## Introduction

Overweight and obesity are major risks for deaths worldwide [1–4] and are estimated to contribute to 44% of the diabetes burden, 23% of the ischaemic heart disease burden and 7–41% of certain cancer burdens [5]. In 2012, it was estimated that 37% of adults (aged 16 and over) were overweight (body mass index (BMI) ≥ 25 to < 30 kg/m^2^) and 25% were classified as obese (BMI ≥ 30 kg/m^2^), based on Health Survey for England data [6]. Data collected between 1993 and 2012 show that the percentage of English adults with a BMI ≥ 18.5 to < 25 kg/m^2^ has decreased from 41 to 32% among men and from 50 to 41% among women [7].

A recent NICE guideline makes recommendations on the provision of weight management services for overweight or obese adults [8]. It recommends that GP practices and other health care professionals who give advice about or refer people to lifestyle weight management programmes should be aware that there should be no upper BMI or upper age limit for funded referrals. However, a number of studies have reported a higher mortality risk associated with weight loss, compared to maintaining a stable weight, particularly in middle-aged and older adults [9–18], although some of these studies did find that gaining weight was also associated with an increased mortality risk [10, 13]. It has been proposed that the observed effects of a higher mortality risk with weight loss may be a balance between the consequences of the loss of potentially harmful abdominal and ectopic fat mass and the loss of potentially beneficial peripheral subcutaneous fat mass and lean body mass [19].

The main objective of this article was to investigate long term mortality from all causes, as well as specifically from cardiovascular disease, cancer and respiratory causes, in relation to measured weight change over an average period of 3.7 years, in 12,580 community-dwelling men and women.

## Methods

### EPIC-Norfolk study design

The Norfolk cohort of the European Prospective Investigation into Cancer and Nutrition (EPIC-Norfolk) is part of the Europe-wide EPIC study, which involves over half a million people in ten countries [20] and was initially planned as a diet and cancer cohort. However, the study in Norfolk broadened its scope from the outset, to investigate the causes of disability and death in middle and later life and to include other lifestyle exposures such as physical activity and psychosocial factors [21]. Participants, aged between 39 and 79 years, were recruited from General Practitioners’ surgeries, based in rural areas of Norfolk and market towns as well as the city of Norwich, from 1993 to 1997. Since virtually all the population of the UK are registered with a general practice through the National Health Service, general practice age sex registers act as a population sampling frame. This cohort at baseline was comparable to the UK national population with regard to many characteristics, including age, sex and anthropometry measurements but it had a lower proportion of current smokers [22].

The study was approved by the Norfolk District Health Authority Ethics Committee and all participants gave written, informed consent.

### Main exposure: weight change

Of the 30,445 men and women, aged 39–79 years, who consented to participate in the study (39% response rate), 25,639 attended a baseline health examination (1HE) between 1993 and 1997 and 15,786 attended a second health examination (2HE) between 1998 and 2000.

At both health examinations, a trained nurse measured weight (to the nearest 0.1 kg) and height (to the nearest 0.1 cm), with participants wearing light clothing and no shoes. Body mass index (BMI) was calculated as weight divided by the square of height (kg/m^2^).

Absolute weight change was calculated as weight (kg) measured at 2HE minus weight (kg) measured at 1HE. Participants were assigned to one of 6 weight change categories: > 5 kg loss, > 2.5–5 kg loss, within 2.5 kg loss or gain (‘maintenance’, considered the reference category), > 2.5–5 kg gain, > 5–10 kg gain, > 10 kg gain.

Annual weight and BMI changes were calculated from the absolute differences in weight and BMI respectively, divided by the participants’ time lapse between the health examinations (kg/year and kg/m^2^/year respectively).

### Participant selection

**Fig. 1:**
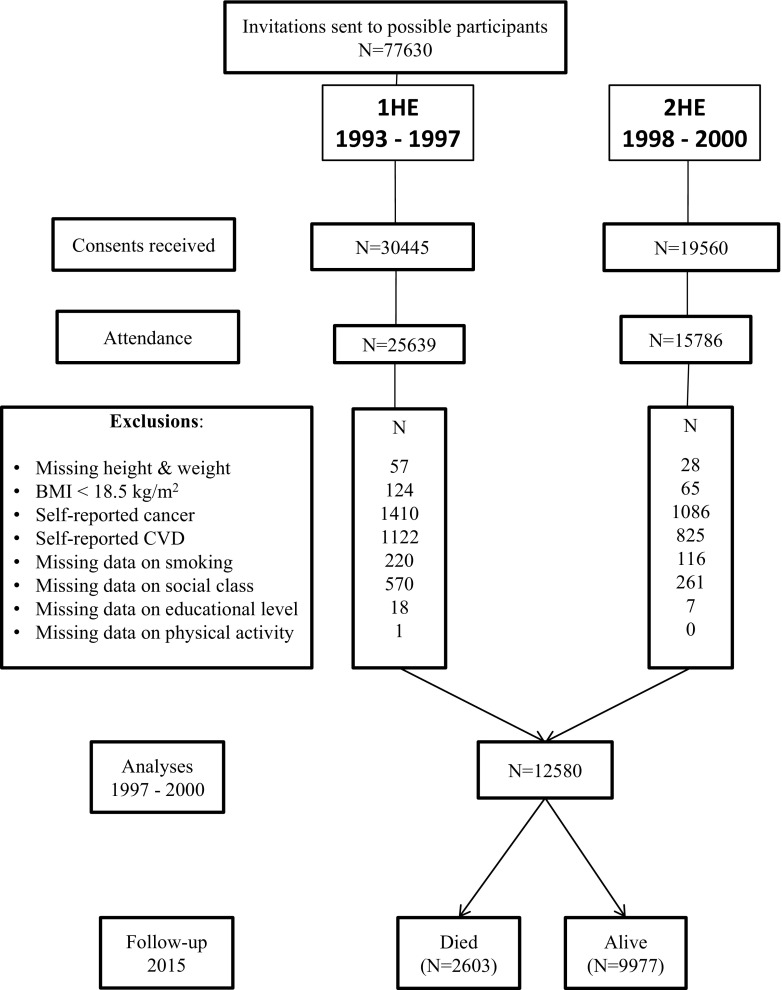
Study population and sample size eligible for mortality analyses

Participants were eligible for inclusion if they had weight and height measurements at both time-points. Participants were excluded from analyses if they had a BMI < 18.5 or who self-reported cancer or cardio-vascular disease (CVD), as were those with missing data on adjustment variables (smoking, social class, educational level and physical activity), in an attempt to address reverse causality. This left 12,580 participants for analyses, out of a maximum of 15,000 for whom we had a weight measurement at both 1HE and 2HE, in order to be able to calculate weight change (Fig. 1).

### Adjustment variables

Data collected via two self-administered Health and Lifestyle Questionnaires (HLQ1 and HLQ2), before the 1HE and 2HE respectively, were used to establish classification of a number of variables. Smoking status (derived from HLQ1 and HLQ2) (never, former, current) was derived from yes and no responses to the following questions “Have you ever smoked as much as one cigarette a day for as long as a year?” and “Do you smoke cigarettes now?”. Self-reported physical activity (derived from HLQ1) was assessed using both occupational and leisure activity and individuals were assigned to one of four categories: inactive, moderately inactive, moderately active and active [23, 24]. Occupational social class at 1HE was defined according to the Registrar General’s classification. Non-manual occupations were represented by codes I, (professional) II, (managerial and technical), IIIa (non-manual skilled) occupations while manual occupations were represented by codes IIIb (manual skilled), IV (partly skilled) and V (unskilled) occupations [25]. Educational level at 1HE was based on the highest qualification attained and was categorised into four groups: degree or equivalent, A level or equivalent, O level or equivalent and less than O level or no qualifications. These four categories correspond to the International Standard Classification of Education (ISCED) 1997 [26] of bachelor/master/doctoral or equivalent (ISCED 5A-6), post-secondary non-tertiary education or short-cycle tertiary education (ISCED 3A-5B), upper secondary education (ISCED 3C-3B) and pre-primary, primary and lower secondary (ISCED 0–2) respectively. In this paper, those with an educational level of O level and above were combined into one category. Participants were asked about their medical histories with the question “Has the doctor ever told you that you have any of the following?” followed by a list of conditions that included heart attack, stroke, cancer, asthma and bronchitis (derived from HLQ1 and HLQ2).

HLQ2 data was used to classify participants regarding recent weight loss, with the question “If you have lost more than 5 kgs (10 lbs) in the last five years, how did this weight loss occur?” Options available included diet, exercise and illness.

### Endpoints

All participants were flagged for death certification up until the end of March 2015, at the Office of National Statistics, United Kingdom. Death certificates were coded by nosologists according to the International Classification of Diseases (ICD). An underlying cause of death was defined by using ICD codes as follows: cancer death (ICD9, 140-208 or ICD10 C00-C97), cardiovascular death (ICD9 400-438 or ICD10 I10-I79), or respiratory disease (ICD9 460-519 or ICD10 J00-J99). Deaths that were not attributable to the three aforementioned causes were classified as deaths from other causes and included deaths from dementia, Parkinson’s disease, bladder and renal diseases.

### Statistical analyses

All adjustment variables were those measured at the first health examination in 1993–1997. The follow-up time was the underlying time variable; median (IQR) follow-up time was 15.4 (14.8, 16.2) years and began at the 2HE. The censor date was the date of death or end of the administrative follow-up (31st March 2015). Characteristics of the study population were summarised by weight change category, using means and SDs for continuous variables and frequencies and percentages for categorical variables. To ascertain whether variables should be included as predictors (with total mortality as the outcome), we performed the log-rank test of equality across strata for all the categorical variables and Cox’s univariate proportional hazards regression for all the continuous variables. The predictors used in the final model were all variables for which the *P* value was < 0.20 in the univariate analyses and for which we also observed an association between the possible confounder and weight change categories. The Cox proportional hazards model was used to determine Hazard Ratios (HR) of all-cause and cause-specific mortality by weight change category for men and women separately, using a series of cumulative adjustment models: age (continuous variable), (model 1); including smoking (categorical variable), (model 2); including BMI (continuous variable), physical activity (4 categories), social class (manual vs. non-manual) and educational level (no qualifications vs. O level and above) (model 3). The interaction between sex and continuous BMI was not found to be significant (*P* = 0.7606). We tested for the proportional hazards assumption by including time interaction variables in the Cox regression models. Age was found to violate our test of the proportional hazards assumption (*P* < 0.0001). However, when we included the time interaction for age, only minimal changes to the hazard ratios of our main exposure (weight change) were observed. The category of greatest weight loss was also found to violate our test of the proportional hazards assumption (*P* < 0.01) and will be discussed later in the manuscript. In sensitivity analyses, we also examined HRs by weight change category, stratified by age and sex, in those who said they had lost weight as a result of dieting and after the exclusion of individuals who died within 3 or 5 years after the second health examination or those who said they had lost weight because of illness, as well as after excluding participants who self-reported asthma or bronchitis at either time-point. The data were analysed using Stata 12 (STATA Corp., Texas, USA).

## Results

### Cohort description

After exclusion, there were 12,580 men and women for analyses (80% of those who had attended 2HE), aged 42–82 years at the 2HE. The mean weight change over the average 4 years between 1HE and 2HE was a gain of 1.29 kg (SD 3.62) in men and 1.39 kg (SD 4.13) in women. Men were in general slightly older than women with a mean age of 62.1 and 61.0 years at 2HE respectively. Men also had a higher mean BMI than women at both health examinations, with 55.4% classified as overweight and 14.5% as obese at 2HE; in women these percentages were 40.4 and 18.4%.

Minimal differences were observed in the baseline characteristics of those who attended both 1HE and 2HE, before and after exclusions were applied (Supplementary Table 1). However, the prevalence of self-reported CVD, cancer, asthma and bronchitis was lower in those who also attended 2HE. Additionally, the percentage of deaths that occurred was lower in both men and women, after exclusion criteria were applied, and the percentage of participants who maintained their weight was slightly higher, which may be indicative of healthy volunteer bias. Nevertheless, the cohort still represents a diverse population with a wide socio-economic distribution and range of lifestyle factors, including physical activity, smoking status and weight.

Table 1 displays descriptive characteristics of men and women, by weight change category. Weight maintenance, which corresponded to a mean annual weight increase of 0.10 kg/year (SD 0.39) in men and 0.11 kg/year (SD 0.40), was observed in 54% of men and 52% of women. Participants with the highest weight gain or loss compared to weight maintenance were those with the highest weight and BMI at 1HE. Current smokers at 2HE were more likely to have lost weight whereas former smokers were more likely to have gained weight. Those who lost weight were more likely to be physically inactive; physically active participants were least likely to have lost weight. Manual workers were more likely to have gained more than 10 kg whereas non-manual workers were more likely to have lost more than 5 kg, compared to maintaining their weight. Women with no qualifications were more likely to have gained more than 10 kg, compared to weight maintenance A higher proportion of women than men said that they had lost weight as a result of dieting (10.9 vs. 4.9% respectively). However, approximately 38% of these participants who said that they had dieted were within 2.5 kg of their baseline weight, while 35% of men and 32% of women had lost more than 2.5 kg and 27% of men and 30% of women had gained more than 2.5 kg from the baseline assessment. Similar percentages of men and women stated that illness was the cause of their weight loss (2.4 vs. 2.5% respectively).

**Table 1:** Characteristics of 5479 men and 7101 women by measured weight change category from 1HE (1993–1997) to 2HE (1998–2000)

	Weight change categories					
	Loss > 5 kg	Loss > 2.5 and ≤ 5 kg	Loss or gain ≤ 2.5 kg	Gain > 2.5 and ≤ 5 kg	Gain > 5 and ≤ 10 kg	Gain > 10 kg
*MEN, N (row%)*	215 (3.9)	423 (7.7)	2983 (54.4)	1206 (22.0)	577 (10.5)	75 (1.4)
Weight (1HE), kg	86.5 (10.7)	81.7 (11.3)	79.1 (10.3)	79.8 (10.5)	82.8 (12.3)	86.3 (13.9)
Weight (2HE), kg	79.0 (10.4)	78.1 (11.2)	79.4 (10.3)	83.4 (10.6)	89.4 (12.4)	99.1 (14.3)
Annual weight change, kg/year	− 2.1 (0.8)	− 1.0 (0.3)	0.1 (0.4)	1.0 (0.3)	1.9 (0.5)	3.5 (1.1)
Age (1HE), years	60.5 (9.2)	60.8 (9.0)	59.6 (8.9)	57.6 (8.8)	56.8 (8.4)	55.9 (8.7)
Age (2HE), years	63.7 (9.2)	64.2 (9.1)	62.7 (9.0)	60.8 (8.9)	60.1 (8.5)	59.2 (8.7)
BMI (1HE), kg/m^2^	28.1 (3.2)	27.0 (3.2)	26.1 (2.9)	26.1 (3.0)	26.8 (3.5)	28.1 (4.6)
BMI (2HE), kg/m^2^	25.8 (3.1)	25.9 (3.2)	26.3 (3.0)	27.3 (3.0)	29.0 (3.6)	32.1 (4.6)
Annual BMI change, kg/m^2^/year	− 0.6 (0.3)	− 0.3 (0.1)	0.1 (0.1)	0.4 (0.1)	0.6 (0.2)	1.1 (0.6)
Smoking status (1HE)						
Current (509, 9.3%)	27 (12.6)	45 (10.6)	228 (7.6)	119 (9.9)	79 (13.7)	11 (14.7)
Former (2902, 53.0%)	106 (49.3)	229 (54.1)	1608 (53.9)	611 (50.7)	307 (53.2)	41 (54.7)
Never (2068, 37.7%)	82 (38.1)	149 (35.2)	1147 (38.4)	476 (39.5)	191 (33.1)	23 (30.7)
Smoking status (2HE)						
Current (440, 8.0%)	28 (13.0)	47 (11.1)	225 (7.5)	93 (7.7)	41 (7.1)	6 (8.0)
Former (2974, 54.3%)	105 (48.8)	227 (53.7)	1613 (54.1)	637 (52.8)	346 (60.0)	46 (61.3)
Never (2065, 37.7%)	82 (38.1)	149 (35.2)	1145 (38.4)	476 (39.5)	190 (32.9)	23 (30.7)
Physical activity (1HE)						
Inactive (1446, 26.4%)	86 (40.0)	128 (30.3)	770 (25.8)	288 (23.9)	154 (26.7)	20 (26.7)
Moderately inactive (1356, 24.8%)	51 (23.7)	105 (24.8)	751 (25.2)	315 (26.1)	120 (20.8)	14 (18.7)
Moderately active (1381, 25.2%)	43 (20.0)	95 (22.5)	746 (25.0)	300 (24.9)	174 (30.2)	23 (30.7)
Active (1296, 23.6%)	35 (16.3)	95 (22.5)	716 (24.0)	303 (25.1)	129 (22.4)	18 (24.0)
Social class (1HE)						
Non-manual (3401, 62.1%)	139 (64.6)	247 (58.4)	1862 (62.4)	766 (63.5)	345 (59.8)	42 (56.0)
Manual (2078, 37.9%)	76 (35.4)	176 (41.6)	1121 (37.6)	440 (36.5)	232 (40.2)	33 (44.0)
Educational level (1HE)						
No qualifications (1447, 26.4%)	60 (27.9)	127 (30.0)	772 (25.9)	302 (25.0)	166 (28.8)	20 (26.7)
O level and above (4032, 73.6%)	155 (72.1)	296 (70.0)	2211 (74.1)	904 (75.0)	411 (71.2)	55 (73.3)
Lost weight in last 5 years (2HE)						
Diet (267, 4.9%)	54 (20.1)	40 (15.3)	102 (38.1)	33 (12.3)	28 (10.4)	10 (3.7)
Illness (130, 2.4%)	22 (16.9)	23 (17.7)	54 (41.5)	19 (14.6)	8 (6.2)	4 (3.1)
*WOMEN, N (row* *%)*	362 (5.1)	517 (7.3)	3690 (52.0)	1540 (21.7)	841 (11.8)	151 (2.1)
Weight (1HE), kg	77.2 (13.3)	69.3 (11.0)	65.8 (10.4)	66.8 (10.4)	70.0 (11.2)	74.1 (12.4)
Weight (2HE), kg	69.0 (12.4)	65.7 (11.1)	66.2 (10.4)	70.4 (10.4)	76.7 (11.4)	87.6 (13.1)
Annual weight change, kg	− 2.4 (1.3)	− 1.0 (0.3)	0.1 (0.4)	1.0 (0.3)	1.9 (0.6)	3.7 (1.5)
Age (1HE), years	58.4 (9.1)	59.5 (9.1)	58.4 (8.9)	57.0 (8.7)	55.8 (8.1)	54.8 (7.1)
Age (2HE), years	61.5 (9.3)	62.6 (9.2)	61.6 (9.0)	60.1 (8.9)	59.1 (8.2)	58.2 (7.3)
BMI (1HE), kg/m^2^	29.3 (4.9)	26.6 (3.9)	25.3 (3.8)	25.6 (3.8)	26.7 (4.1)	27.9 (4.4)
BMI (2HE), kg/m^2^	26.4 (4.6)	25.4 (3.9)	25.6 (3.9)	27.1 (3.8)	29.4 (4.2)	33.2 (4.9)
Annual BMI change, kg/m^2^/year	− 0.8 (0.5)	− 0.3 (0.1)	0.1 (0.2)	0.4 (0.1)	0.7 (0.2)	1.4 (0.6)
Smoking status (1HE)						
Current (641, 9.0%)	35 (9.7)	53 (10.2)	296 (8.0)	142 (9.2)	96 (11.4)	19 (12.6)
Former (2239, 31.5%)	132 (36.5)	166 (32.1)	1129 (30.6)	478 (31.0)	282 (33.5)	52 (34.4)
Never (4221, 59.4%)	195 (53.9)	298 (57.6)	2265 (61.4)	920 (59.7)	463 (55.0)	80 (53.0)
Smoking status (2HE)						
Current (569, 8.0%)	32 (8.8)	56 (10.8)	280 (7.6)	117 (7.6)	74 (8.8)	10 (6.6)
Former (2317, 32.6%)	135 (37.3)	163 (31.5)	1150 (31.2)	504 (32.7)	304 (36.2)	61 (40.4)
Never (4215, 59.4%)	195 (53.9)	298 (57.6)	2260 (61.2)	919 (59.7)	463 (55.0)	80 (53.0)
Physical activity (1HE)						
Inactive (1761, 24.8%)	115 (31.8)	145 (28.0)	906 (24.6)	354 (23.0)	201 (23.9)	40 (26.5)
Moderately inactive (2360, 33.2%)	119 (32.9)	168 (32.5)	1228 (33.3)	518 (33.6)	275 (32.7)	52 (34.4)
Moderately active (1722, 24.2%)	82 (22.6)	117 (22.6)	896 (24.3)	392 (25.4)	202 (24.0)	33 (21.8)
Active (1258, 17.7%)	46 (12.7)	87 (16.8)	660 (17.9)	276 (17.9)	163 (19.4)	26 (17.2)
Social class (1HE)						
Non-manual (4502, 63.4%)	222 (61.3)	339 (65.6)	2395 (64.9)	933 (60.6)	532 (63.3)	81 (53.6)
Manual (2599, 36.6%)	140 (38.7)	178 (34.4)	1295 (35.1)	607 (39.4)	309 (36.7)	70 (46.4)
Educational level (1HE)						
No qualifications (2598, 36.6%)	142 (39.2)	183 (35.4)	1356 (36.8)	554 (36.0)	296 (35.2)	67 (44.4)
O level and above (4503, 63.4%)	220 (60.8)	334 (64.6)	2334 (63.2)	986 (64.0)	545 (64.8)	84 (55.6)
Lost weight in last 5 years (2HE)						
Diet (777, 10.9%)	129 (16.6)	118 (15.5)	296 (37.9)	115 (14.8)	83 (10.5)	36 (4.6)
Illness (175, 2.5%)	31 (18.2)	23 (12.7)	67 (38.7)	30 (17.1)	17 (9.4)	7 (3.9)

Continuous variables are Mean (SD) and categorical variables are n (%)

*1HE* 1st health examination, *2HE* 2nd health examination, *BMI* body mass index

### Main analyses: all-cause and cause-specific mortality

Over a median follow-up period of 15 years, 1421 deaths in men were recorded (401 deaths from CVD, 539 cancer-related deaths, 135 deaths from respiratory diseases and 346 deaths from other causes).

Total and cause-specific mortality HRs by weight change category for men are shown in Table 2. Men who lost weight had a statistically significant higher hazard of all-cause mortality than those who maintained their weight (HR 1.83 (CI 1.47–2.29) in those who lost more than 10 kg and 1.29 (CI 1.09–1.54) in those who lost between 2.5 and 5 kg); those who gained more than 10 kg also had a higher hazard but this was not significant. The findings for CVD mortality in men were stronger than for all-cause mortality. In model 3, adjusting for age, smoking, BMI, physical activity, social class and educational level, men who had a weight loss greater than 5 kg had more than double the hazard of CVD mortality, compared to those who maintained their weight [HR 2.09 (CI 1.41–3.09)]. Borderline significant findings for cancer mortality in men were found in those who lost the greatest amount of weight [HR 1.45 (CI 0.98–2.15)] whereas those who gained between 5 and 10 kg had a significantly lower hazard of 0.70 (CI 0.49–0.99). Gaining or losing more than 5 kg was significantly associated with a higher hazard of death from respiratory causes. In model 3, men who lost more than 5 kg had a HR of dying from respiratory causes of 4.50 (CI 2.51–8.08) whereas those who gained more than 10 kg had a HR of 3.97 (CI 1.43–11.01). After the exclusion of participants who self-reported having asthma or bronchitis at either time-point, the HR in men who lost more than 5 kg minimally attenuated to 4.08 (CI 2.00–8.33); in those who gained more than 10 kg, the HR was 2.71 (0.65–11.24) (data not shown). No significant findings were found with regard to weight change and dying from other causes in men. In general, the addition of BMI, physical activity, social class and educational level to the models had minimal effects on the HRs. Adjusting for categories of BMI, rather than as a continuous variable minimally changed the weight change-mortality associations.

**Table 2:** Total and cause-specific mortality in 5479 men by weight change category

	Weight change categories					
	Loss > 5 kg	Loss > 2.5 and ≤ 5 kg	Loss or gain ≤ 2.5 kg	Gain > 2.5 and ≤ 5 kg	Gain > 5 and ≤ 10 kg	Gain > 10 kg
Men, N	215	423	2983	1206	577	75
All cause mortality						
Number of events (%)	91 (41.9)	154 (36.2)	801 (26.5)	259 (21.1)	128 (21.9)	20 (26.3)
Model 1	*** 1.96 (1.57–2.44)	** 1.36 (1.14–1.62)	Ref	0.96 (0.83–1.11)	1.09 (0.90–1.32)	* 1.66 (1.06–2.59)
Model 2	*** 1.93 (1.55–2.40)	** 1.31 (1.10–1.56)	Ref	0.94 (0.81–1.08)	1.02 (0.84–1.23)	1.49 (0.95–2.32)
Model 3	*** 1.83 (1.46–2.29)	** 1.29 (1.09–1.54)	Ref	0.94 (0.81–1.08)	1.01 (0.84–1.23)	1.49 (0.95–2.33)
CVD mortality						
Number of events (%)	31 (14.3)	47 (11.0)	218 (7.2)	77 (6.3)	34 (5.8)	7 (9.2)
Model 1	*** 2.46 (1.68–3.61)	* 1.52 (1.10–2.09)	Ref	1.07 (0.82–1.39)	1.14 (0.80–1.65)	* 2.26 (1.06–4.80)
Model 2	*** 2.44 (1.66–3.58)	* 1.47 (1.07–2.02)	Ref	1.04 (0.80–1.36)	1.07 (0.74–1.54)	2.02 (0.95–4.31)
Model 3	*** 2.09 (1.41–3.09)	* 1.41 (1.02–1.94)	Ref	1.06 (0.81–1.38)	1.05 (0.73–1.51)	1.90 (0.89–4.07)
Cancer mortality						
Number of events (%)	28 (12.9)	53 (12.4)	310 (10.2)	112 (9.1)	39 (6.7)	8 (10.5)
Model 1	* 1.52 (1.03–2.24)	1.19 (0.88–1.60)	Ref	1.02 (0.82–1.27)	0.75 (0.53–1.06)	1.53 (0.76–3.09)
Model 2	* 1.50 (1.02–2.21)	1.15 (0.86–1.54)	Ref	1.00 (0.80–1.24)	* 0.70 (0.50–0.99)	1.37 (0.68–2.76)
Model 3	1.45 (0.98–2.15)	1.14 (0.84–1.52)	Ref	1.01 (0.80–1.25)	* 0.70 (0.49–0.99)	1.34 (0.66–2.72)
Respiratory mortality						
Number of events (%)	15 (6.9)	16 (3.8)	66 (2.2)	18 (1.5)	18 (3.1)	4 (5.3)
Model 1	*** 4.35 (2.48–7.64)	* 1.74 (1.01–3.02)	Ref	0.85 (0.50–1.46)	** 2.14 (1.26–3.61)	** 4.61 (1.68–12.68)
Model 2	*** 4.22 (2.40–7.43)	1.59 (0.92–2.75)	Ref	0.80 (0.47–1.38)	* 1.80 (1.06–3.05)	* 3.56 (1.29–9.82)
Model 3	*** 4.50 (2.51–8.08)	1.62 (0.93–2.82)	Ref	0.81 (0.47–1.38)	** 1.82 (1.08–3.10)	** 3.97 (1.43–11.01)
Other cause mortality						
Number of events (%)	17 (7.8)	38 (8.9)	207 (6.8)	52 (4.2)	37 (6.3)	1 (1.3)
Model 1	1.42 (0.85–2.36)	1.33 (0.94–2.36)	Ref	0.77 (0.57–1.05)	1.29 (0.90–1.83)	0.34 (0.05–2.44)
Model 2	1.40 (0.84–2.32)	1.30 (0.92–1.84)	Ref	0.76 (0.56–1.04)	1.24 (0.87–1.77)	0.33 (0.04–2.34)
Model 3	1.41 (0.84–2.36)	1.30 (0.92–1.85)	Ref	0.75 (0.55–1.02)	1.25 (0.88–1.78)	0.34 (0.05–2.39)

Associations were assessed using Cox proportional hazards regression with a median follow-up from 2HE of 15 years. Results are hazard ratios and 95% confidence intervals, HR (95% CI)

Model 1: adjusted for age (continuous)

Model 2: Model 1 + further adjusted for smoking (categorical)

Model 3: Model 2 + further adjusted for BMI (continuous), physical activity (categorical), social class (categorical) and educational level (categorical)

*2HE* 2nd health examination *CVD* cardiovascular disease, *BMI* body mass index

Significance of HRs: ***P < 0.001; **P < 0.01; *P < 0.05

In women, 1182 deaths were recorded over a median follow-up period of 15 years, (348 deaths from CVD, 442 cancer-related deaths, 91 deaths from respiratory causes and 301 deaths from other causes). Table 3 presents data on total and cause-specific mortality HRs for women, by weight change category. Women who lost more than 5 kg had a significantly higher hazard of all-cause mortality of 1.68 (CI 1.34–2.10) compared to those who maintained their weight, whilst those who lost between 2.5 and 5 kg had a HR of 1.32 (CI 1.09–1.60). Losing weight and gaining more than 5 kg was associated with a higher hazard for CVD mortality, but these findings were not significant, although a weight loss of more than 5 kg was borderline significant [HR 1.54 (CI 1.00–2.37)]. Regarding cause-specific mortality in women who lost more than 5 kg, only respiratory deaths and other causes of deaths were significant in model 3. After the exclusion of participants who self-reported having asthma or bronchitis at either time-point, this HR in those who lost more than 5 kg was no longer significant [HR 1.78, (CI 0.68–4.69)]. In those who lost more than 5 kg, the hazard for deaths from other causes was 2.17 (CI 1.44–3.27). In general, the addition of BMI, physical activity, social class and educational level to the models had minimal effects on the HRs. Adjusting for categories of BMI instead of using BMI as a continuous variable minimally changed the observed associations between weight change and mortality. See Supplementary Table 2 for the full coefficient tables of model 3 for all-cause mortality in men and women.

**Table 3:** Total and cause-specific mortality in 7101 women by weight change category

	Weight change categories					
	Loss > 5 kg	Loss > 2.5 and ≤ 5 kg	Loss or gain ≤ 2.5 kg	Gain > 2.5 and ≤ 5 kg	Gain > 5 and ≤ 10 kg	Gain > 10 kg
Women, N	362	517	3690	1540	841	151
All cause mortality						
Number of events (%)	94 (25.4)	128 (24.3)	651 (17.3)	223 (14.2)	115 (13.5)	15 (9.8)
Model 1	*** 1.77 (1.42–2.20)	** 1.34 (1.11–1.63)	Ref	0.94 (0.81–1.10)	1.14 (0.93–1.39)	1.06 (0.63–1.77)
Model 2	*** 1.72 (1.38–2.15)	** 1.32 (1.08–1.59)	Ref	0.94 (0.80–1.09)	1.12 (0.91–1.37)	1.02 (0.61–1.70)
Model 3	*** 1.68 (1.34–2.10)	** 1.32 (1.09–1.60)	Ref	0.93 (0.80–1.09)	1.11 (0.90–1.36)	0.98 (0.58–1.64)
CVD mortality						
Number of events (%)	25 (6.8)	33 (6.3)	196 (5.2)	70 (4.5)	33 (3.9)	6 (3.9)
Model 1	* 1.56 (1.02–2.39)	1.09 (0.75–1.59)	Ref	0.97 (0.73–1.28)	1.24 (0.86–1.80)	1.84 (0.81–4.17)
Model 2	* 1.55 (1.01–2.37)	1.08 (0.74–1.57)	Ref	0.96 (0.72–1.28)	1.23 (0.85–1.79)	1.83 (0.81–4.16)
Model 3	1.54 (1.00–2.37)	1.10 (0.75–1.60)	Ref	0.97 (0.73–1.29)	1.24 (0.85–1.80)	1.75 (0.77–4.00)
Cancer mortality						
Number of events (%)	30 (8.1)	47 (8.9)	234 (6.2)	90 (5.7)	48 (5.6)	5 (3.3)
Model 1	* 1.53 (1.04–2.23)	1.38 (1.00–1.91)	Ref	1.01 (0.79–1.29)	1.14 (0.83–1.56)	0.72 (0.30–1.76)
Model 2	* 1.48 (1.01–2.16)	1.35 (0.98–1.86)	Ref	0.99 (0.78–1.27)	1.10 (0.81–1.51)	0.68 (0.28–1.66)
Model 3	1.36 (0.92–2.01)	1.33 (0.96–1.83)	Ref	0.98 (0.76–1.25)	1.07 (0.78–1.47)	0.64 (0.26–1.55)
Respiratory mortality						
Number of events (%)	11 (3.0)	12 (2.3)	50 (1.3)	13 (0.8)	8 (0.9)	1 (0.6)
Model 1	* 2.34 (1.15–4.77)	1.56 (0.83–2.94)	Ref	0.74 (0.40–1.37)	1.04 (0.47–2.31)	1.26 (0.17–9.22)
Model 2	* 2.28 (1.18–4.65)	1.52 (0.81–2.85)	Ref	0.74 (0.40–1.36)	1.02 (0.46–2.27)	1.22 (0.17–8.98)
Model 3	* 2.30 (1.11–4.74)	1.55 (0.82–2.91)	Ref	0.75 (0.40–1.38)	1.04 (0.47–2.31)	1.25 (0.17–9.24)
Other cause mortality						
Number of events (%)	28 (7.6)	36 (6.8)	171 (4.6)	50 (3.2)	26 (3.1)	3 (2.0)
Model 1	*** 2.21 (1.48–3.31)	* 1.49 (1.04–2.14)	Ref	0.87 (0.63–1.19)	1.06 (0.69–1.61)	0.98 (0.31–3.09)
Model 2	*** 2.17 (1.45–3.25)	* 1.47 (1.02–2.11)	Ref	0.86 (0.63–1.18)	1.04 (0.68–1.59)	0.95 (0.30–3.00)
Model 3	*** 2.17 (1.44–3.27)	* 1.48 (1.03–2.12)	Ref	0.86 (0.63–1.19)	1.04 (0.68–1.60)	0.95 (0.30–3.00)

Associations were assessed using Cox proportional hazards regression with a median follow-up from 2HE of 15 years. Results are hazard ratios and 95% confidence intervals, HR (95% CI)

Model 1: adjusted for age (continuous)

Model 2: Model 1 + further adjusted for smoking (categorical)

Model 3: Model 2 + further adjusted for BMI (continuous), physical activity (categorical), social class (categorical) and educational level (categorical)

*2HE* 2nd health examination *CVD* cardiovascular disease, *BMI* body mass index

Significance of HRs: ***P < 0.001; **P < 0.01; *P < 0.05

### Sensitivity analyses

HRs for all-cause mortality, adjusted for age, sex, BMI, physical activity, smoking status, social class and educational level, and by stratified variables per weight change category are shown in Table 4.

**Table 4:** Cox multivariable-adjusted HRs^a^ after 15 years of follow-up for all-cause mortality in 12,580 men and women

				Weight change categories					
				Loss > 5 kg	Loss > 2.5 and ≤ 5 kg	Loss or gain ≤ 2.5 kg	Gain > 2.5 and ≤ 5 kg	Gain > 5 and ≤ 10 kg	Gain > 10 kg
Events (n)/N				180/577	277/940	1407/6673	466/2746	238/1418	35/226

	N	%	Events	HR (95% CI)	HR (95% CI)		HR (95% CI)	HR (95% CI)	HR (95% CI)
All	12,580	100.0	2603	*** 1.74 (1.48–2.03)	*** 1.31 (1.15–1.49)	Ref	0.93 (0.84–1.04)	1.05 (0.91–1.21)	1.21 (0.86–1.69)
*By sex*									
Men	5479	43.6	1421	*** 1.83 (1.46–2.29)	** 1.29 (1.09–1.54)	Ref	0.94 (0.81–1.08)	1.01 (0.84–1.23)	1.49 (0.95–2.33)
Women	7101	56.4	1182	*** 1.68 (1.34–2.10)	** 1.32 (1.09–1.60)	Ref	0.93 (0.80–1.09)	1.11 (0.90–1.36)	0.98 (0.58–1.64)
*By age (1HE)*									
<65 years old	9251	73.5	982	*** 1.64 (1.26–2.15)	** 1.36 (1.08–1.70)	Ref	0.91 (0.77–1.08)	1.07 (0.88–1.30)	1.09 (0.70–1.72)
≥65 years old	3329	26.5	1621	*** 1.80 (1.48–2.19)	** 1.28 (1.10–1.51)	Ref	0.95 (0.83–1.09)	1.02 (0.84–1.24)	1.37 (0.82–2.29)
*By age (2HE)*									
<65 years old	7734	61.5	619	** 1.67 (1.20–2.32)	1.26 (0.94–1.69)	Ref	0.90 (0.73–1.10)	1.04 (0.81–1.33)	0.76 (0.42–1.40)
≥65 years old	4846	38.5	1984	*** 1.73 (1.45–2.08)	*** 1.29 (1.12–1.50)	Ref	0.94 (0.84–1.07)	1.03 (0.87–1.22)	* 1.51 (1.01–2.27)
*By smoking status (1HE)*									
Current	1150	9.1	318	* 1.67 (1.10–2.54)	1.25 (0.89–1.76)	Ref	0.73 (0.54–1.00)	0.82 (0.57–1.19)	0.80 (0.33–1.98)
Former	5141	40.9	1293	** 1.54 (1.20–1.96)	*** 1.41 (1.17–1.68)	Ref	0.95 (0.82–1.10)	1.06 (0.87–1.29)	1.23 (0.78–1.95)
Never	6289	50.0	992	*** 1.98 (1.56–2.53)	1.19 (0.95–1.48)	Ref	0.98 (0.83–1.16)	1.14 (0.90–1.43)	1.44 (0.78–2.62)
*By BMI (1HE)*									
≥ 18.5 to < 25 kg/m^2^	5288	42.0	973	** 1.76 (1.23–2.50)	** 1.43 (1.15–1.79)	Ref	0.93 (0.79–1.10)	0.96 (0.75–1.23)	1.07 (0.53–2.16)
≥ 25 to < 30 kg/m^2^	5630	44.8	1226	*** 1.84 (1.47–2.30)	* 1.25 (1.04–1.50)	Ref	0.94 (0.81–1.10)	1.02 (0.83–1.25)	1.43 (0.88–2.32)
≥ 30 kg/m^2^	1662	13.2	404	** 1.56 (1.15–2.12)	1.33 (0.96–1.86)	Ref	0.90 (0.67–1.22)	1.24 (0.92–1.67)	1.02 (0.54–1.95)
*By BMI (2HE)*									
≥ 18.5 to < 25 kg/m^2^	4578	36.4	878	*** 1.82 (1.45–2.30)	* 1.28 (1.05–1.55)	Ref	0.92 (0.74–1.13)	0.72 (0.46–1.11)	4.84 (0.67–34.69)
≥ 25 to < 30 kg/m^2^	5900	46.9	1246	*** 1.75 (1.36–2.24)	** 1.30 (1.07–1.58)	Ref	0.95 (0.82–1.10)	1.06 (0.87–1.30)	0.92 (0.43–1.94)
≥ 30 kg/m^2^	2102	16.7	479	* 1.56 (1.02–2.40)	1.42 (0.98–2.06)	Ref	0.91 (0.72–1.16)	1.05 (0.82–1.35)	1.08 (0.71–1.65)
*By physical activity (1HE)*									
Inactive	3207	25.6	964	*** 1.82 (1.44–2.30)	*** 1.46 (1.18–1.79)	Ref	1.10 (0.92–1.30)	1.04 (0.82–1.32)	1.62 (0.96–2.72)
Moderately inactive	3716	29.5	723	*** 1.84 (1.35–2.51)	1.21 (0.94–1.56)	Ref	0.94 (0.78–1.15)	1.21 (0.93–1.58)	1.35 (0.72–2.55)
Moderately active	3103	24.7	520	* 1.48 (1.01–2.16)	1.28 (0.96–1.71)	Ref	* 0.75 (0.59–0.96)	0.89 (0.66–1.20)	0.80 (0.36–1.80)
Active	2554	20.3	396	* 1.72 (1.02–2.87)	1.18 (0.85–1.65)	Ref	0.82 (0.62–1.07)	1.04 (0.73–1.48)	0.82 (0.30–2.23)
*By social class (1HE)*									
Non-manual	7903	62.8	1610	*** 1.85 (1.52–2.25)	1.14 (0.96–1.36)	Ref	0.94 (0.83–1.08)	1.02 (0.85–1.21)	1.34 (0.87–2.07)
Manual	4677	37.2	993	** 1.55 (1.18–2.02)	*** 1.60 (1.32–1.95)	Ref	0.93 (0.78–1.10)	1.09 (0.87–1.36)	1.05 (0.61–1.79)
*By education level (1HE)*									
No qualifications	4045	32.2	1089	*** 1.77 (1.39–2.26)	* 1.26 (1.02–1.54)	Ref	0.92 (0.79–1.08)	1.12 (0.91–1.38)	1.35 (0.83–2.20)
O level and above	8535	67.8	1514	*** 1.72 (1.40–2.12)	** 1.33 (1.13–1.58)	Ref	0.95 (0.82–1.09)	1.01 (0.84–1.21)	1.08 (0.67–1.73)
*Excluding early deaths*									
Events (n)/N				160/557	257/920	1302/6568	440/2720	221/1401	31/222
Excluding deaths < 3 years	12,388	98.5	2411	*** 1.70 (1.44–2.01)	*** 1.32 (1.16–1.51)	Ref	0.95 (0.86–1.06)	1.06 (0.91–1.22)	1.16 (0.81–1.67)
Events (n)/N				135/532	231/894	1190/6456	401/2681	202/1382	28/219
Excluding deaths < 5 years	12,164	96.7	2187	*** 1.61 (1.35–1.94)	*** 1.31 (1.14–1.51)	Ref	0.95 (0.85–1.06)	1.06 (0.91–1.23)	1.17 (0.80–1.70)
Events (n)/N				151/524	263/894	1364/6552	452/2697	232/1393	32/215
Excluding persons who said they had lost weight due to illness	12,275	97.6	2494	*** 1.61 (1.36–1.91)	*** 1.32 (1.15–1.50)	Ref	0.94 (0.84–1.04)	1.05 (0.92–1.21)	1.18 (0.83–1.67)
Events (n)/N				46/183	37/158	57/398	19/148	20/111	4/46
Persons who said they had lost weight due to dieting N (%)	1044	8.3	183	1.48 (0.99–2.22)	1.40 (0.92–2.13)	Ref	0.78 (0.46–1.33)	* 1.92 (1.14–3.23)	0.96 (0.35–2.67)
Events (n)/N				135/464	223/763	1084/5298	379/2266	176/1120	28/172
Excluding self-reported asthma and bronchitis at 1HE and 2HE N (%)	10,083	80.2	2025	*** 1.71 (1.42–2.05)	*** 1.35 (1.17–1.56)	Ref	0.95 (0.84–1.07)	1.04 (0.88–1.22)	1.32 (0.91–1.93)

Results are given for stratified variables by weight change category

*1HE* 1st health examination, *2HE* 2nd health examination, *BMI* body mass index

^a^Adjusted for age, sex, BMI, physical activity, smoking, social class and educational level (except where the categorical variable was used for stratification)

Significance of HRs: ***P < 0.001; **P < 0.01; *P < 0.05

Similar higher total mortality HRs were found for both men and women who lost weight. Compared to a stable weight, women who gained more than 10 kg had a HR of 0.98 (CI 0.58–1.64) whereas in men, the hazard was higher [1.49 (CI 0.95–2.33)]. We observed similar associations among participants who were younger or older than 65 years of age, whether categorised using 1HE or 2HE data in all weight change categories, with the exception of the greatest weight gain category using 2HE data [the latter probably due to the low number of events and/or participants in respective strata (11/173 and 24/53), which resulted in wide confidence intervals]. Higher hazards were observed in all BMI classifications at both 1HE and 2HE in those who lost weight compared to weight maintenance. In all four physical activity categories, HRs for total mortality were all of a similar direction for weight loss.

HRs for all-cause mortality and weight loss were generally consistent in the three categories of smoking status. However, there was no significant higher risk of mortality in either current or never smokers who lost < 5 kg. Data on the number of cigarettes smoked was available for 1013 of the 1150 current smokers at 1HE and additionally adjusting for this did not modify the association among smokers. We further examined the association of all-cause mortality with weight change, taking into account changes in smoking status (Supplementary Table 3). The greatest weight gain was observed among those participants who reported to have stopped smoking between 1HE and 2HE. Exclusion of recent smokers from the current smokers at 2HE strengthened the all-cause mortality HRs in the greatest weight loss category (HR 1.41 CI 0.92–2.18) (data not shown). Minimal changes were observed in the HRs of former smokers, after the exclusion of those who had recently stopped smoking.

We observed similar associations among participants regarding social class and educational level, in all weight change categories, with those who lost more than 5 kg having significantly higher HRs for all-cause mortality.

Even after excluding participants who died within 3 or 5 years of the 2HE and those who said they had lost weight because of illness, participants who lost weight had significantly higher HRs for all-cause mortality than those who maintained their weight. The observed higher HRs for all-cause mortality in those who had lost weight remained consistent after excluding participants who self-reported having asthma and/or bronchitis at either time-point. We then ran this analysis for respiratory mortality, rather than all-cause mortality, and found that those who lost more than 5 kg had a HR of 2.89 (CI 1.64–5.12), compared to those who maintained their weight; those who lost between 2.5 and 5 kg had a HR of 1.40 (CI 0.84–2.36).

In Supplementary Table 4, we further examined models 2 and 3 for all-cause mortality in both men and women, replacing smoking status at 1HE with smoking history, as categorised in Supplementary Table 3, and observed minimal changes in the HRs.

### Time-varying analysis

When we included a time-interaction variable with weight change, the HR in those who lost more than 5 kg increased from 1.7 to 3.25 (*P* = 0.001) and the HR for follow-up time was 0.73, i.e., participants had a threefold hazard compared to participants who maintained their weight within a year from 2HE; however, this hazard decreased by 27% with every year of follow-up. This decrease in the hazard during follow-up might be explained by misclassification over time of participants with regard to exposure. It is also plausible that some participants lost weight because they were (acutely) ill and therefore had a higher hazard of dying at the start of follow-up.

## Discussion

### Summary of main findings

Findings from this population-based cohort study of 12,580 middle-aged and elderly men and women suggest that weight loss, over the previous 4 years or so, is associated with higher mortality over the next 15 years of follow-up. This result was observed after excluding those who were underweight or who self-reported cancer or CVD, at either time-point. This association was also evident in subgroups of the population, after stratification for age, smoking, BMI, physical activity and the exclusion of individuals who said they had lost weight due to illness and deaths within the first 5 years of follow-up, as well as in dieters who reported to have lost more than 5 kg. Results for weight gain were inconclusive.

### Strengths and limitations

The major strengths of our study include its prospective design, its large population of free-living, middle-aged and elderly men and women, long follow-up time and the availability of information on a large number of factors associated with weight change. In addition, height and weight were objectively measured rather than self-reported and were available at both time-points. In an effort to address reverse causality, we excluded participants with self-reported cancer or CVD, in addition to those who had a BMI < 18.5 kg/m^2^, at either time-point. In our subgroup analyses, we excluded deaths within the first 3 and 5 years, in addition to those who said they had lost weight due to illness.

The main limitations of our cohort study include self-reported disease history, healthy volunteer bias and attrition. It is likely that some individuals in our study had an underlying disease condition that they did not report which may have resulted in weight loss and subsequent death. Whilst the more frail participants may not have returned for the 2HE and/or tended to have been excluded and therefore be under-represented in our analyses, it is plausible that selective attrition of the frailest participants, may have led to an under-estimation of our findings. However, it is also possible that participants in the weight loss categories were the more frail study participants, who were pre-frail at 2HE. The inability to take into account all changes in behaviours, including physical activity during follow-up time is also a limitation, which may have resulted in misclassification of individuals, with subsequent effects on observed associations. Our findings relate solely to changes in weight and not any other anthropometric measurements, such as height, waist circumference, waist–hip ratio, fat mass or muscle mass.

### Comparison of cause-specific mortality with other studies

Regarding CVD mortality, statistically significant associations, after multi-variate adjustment, were found in men who lost weight, but not in women; statistical power was more limited due to the lower number of CVD deaths in women, although the HR in those who lost more than 5 kg suggests a higher hazard compared to stable weight. A prospective study in 5608 middle-aged men by Wannamethee et al. [12] found that sustained weight loss was associated with significantly higher total and CVD mortality, even after adjustment for lifestyle factors and pre-existing diseases and ill-health. Results from the Melbourne Collaborative Cohort Study [9] illustrate that weight loss in men and women, compared to minimal weight increase, was associated with a higher risk of all-cause and CVD mortality. Adams et al. [27] also found that weight loss was associated with a higher hazard ratio for CVD mortality in those aged 50–69 years [HR 1.51 (CI 1.35–1.69)].

We observed higher hazards of cancer mortality in both men and women who lost weight, although these did not quite reach significance. The Melbourne Collaborative Cohort Study [9] concluded that a change in body weight was not associated with obesity-related cancer mortality but the small number of cancer-related deaths in their study may explain why no association was observed. However, three prospective cohort studies found a positive association between cancer mortality and weight loss [28–30], although two of these studies were carried out in Japanese men and women [29, 30] and may not be generalizable to other populations as Japanese obesity rates differ substantially from those of Western populations [31] as do their cancer incidence and mortality rates and major cancer types [32].

In men, there was a significantly higher hazard of dying from respiratory causes in those who lost or gained more than 5 kg, whereas a higher hazard was only found in women who lost more than 5 kg. Attention must be drawn to the low numbers of deaths due to respiratory causes, particularly in men who gained more than 10 kg (n = 4). After the exclusion of participants who self-reported having asthma or bronchitis at either time-point, the HRs of dying from respiratory causes in those who gained more than 5 kg attenuated and were no longer significant. It is well-known that underweight individuals have an increased risk of dying from chronic respiratory disease [33–35]. To address reverse causation, we excluded all underweight participants (BMI < 18.5) at both time-points, in addition to deaths within the first 5 years. In our subgroup analyses, we showed that weight loss was still associated with higher hazards for all-cause mortality, even after excluding participants with prevalent respiratory disease at either time-point. However, it is possible that our exclusion period of deaths within 5 years is too short [35]. The Prospective Cohort Studies Collaboration of 900,000 adults found that each 5-unit lowering in BMI from 25 to 15 kg/m^2^ was associated with a 1.7-fold increase in respiratory mortality [1].

Women who lost more than 2.5 kg had a higher risk of dying from other causes. We cannot be certain that all relevant confounders have been addressed nor that unmeasured confounding is not an issue. It is plausible that this may be explained by undiagnosed pre-existing diseases not explained by the exclusion factors applied. Further disaggregation of this miscellaneous category into more specific disease types may help clarify these results.

### Explanatory factors of all-cause mortality compared to other studies

In our subgroup analyses, we found that both younger [< 65 years at 1HE (73.5% of study population)] and older participants (≥ 65 years) who lost weight had similar higher hazards for total mortality compared to weight maintenance. These findings are in agreement with recent studies that have found higher total mortality risks with weight loss in middle-aged and elderly populations [9, 18].

We observed higher hazards in all BMI classifications at both 1HE and 2HE in those who lost weight compared to weight maintenance. In a stratified analysis, we observed that being obese at 1HE was associated with a higher hazard of death [1.16 (CI 1.03–1.30)] but not being overweight [0.94 (CI 0.86–1.02)] compared to normal weight (data not shown). Our results are therefore not different from the general assumption that being obese is associated with higher mortality [36, 37]. However, weight loss is an additional factor in this association and potentially an effect modifier. In this population-based cohort study, it seems that weight loss when obese is less hazardous than when overweight or normal weight. Controversy has, until recently, surrounded weight loss therapies in obese older adults [38, 39]. However, evidence from randomised controlled trials have reported positive outcomes on physical function, muscle quality and inflammatory status [40, 41].

When studying weight change, smoking is seen as an important source of confounding [42]. Previous studies investigating weight loss and mortality have therefore tended either to restrict analyses to never-smokers or have adjusted for smoking status. The rationale for this is that smokers tend to weigh less than non-smokers but have considerably higher mortality rates [43]. Some controversy surrounds the association between smoking and weight change; in our study, current smokers at 1HE had a greater mean increase in weight than either former or never smokers (1.59, 1.35 and 1.29 kg respectively), which is in agreement with recent studies [44, 45] but prospective investigations performed on three separate large US cohorts found that current non-obese smokers lost weight over a 4-year period [46].

In our analyses, we included smoking status in our multivariate-adjusted models but also stratified by smoking status. In the stratified analysis, we observed higher hazards for all-cause mortality with weight loss in never, former and current smokers, although the HRs were only significant in all three categories when weight loss was greater than 5 kg, suggesting that weight loss greater than 5 kg in this population, was positively associated with higher all-cause mortality, irrespective of smoking status.

There is a wealth of information on weight change and cessation of smoking [47, 48]. Whilst this study was not designed to investigate weight change in relation to changes in smoking status, we found that when we did so, that the greatest mean increase in weight was found in those who had recently stopped smoking (mean = 3.4 kg, SD = 4.8) and that long-term smokers had actually a smaller weight increase than long-term former smokers and never smokers (1.1, 1.4 and 1.3 kg respectively). Those who had recently started smoking had the smallest mean weight increase (0.2 kg). We have shown that our association of higher all-cause mortality with weight loss was strengthened in smokers (2HE) after the removal of recent smokers and that the exclusion of those who had recently stopped smoking only minimally affected the HRs in former smokers. We reran analyses for all-cause mortality, replacing smoking status at 2HE with smoking history, as classified in Supplementary Table 3 and observed minimal changes to the HR (Supplementary Table 4).

Associations between all-cause mortality and weight loss also remained when the data were investigated by categories of physical activity, with those who were less active tending to have slightly higher HRs than more active participants. The inactive category who gained weight had minimally higher mortality hazards but HRs were not significant. However, a large, European, prospective cohort study of 288,498 men and women, which included EPIC-Norfolk participants, found that baseline self-reported physical activity was not associated with a change in body weight in men or women, after adjustment for confounders and suggested that the association between lower physical activity and a gain in body weight may be restricted to younger and normal-weight individuals [49]. Only data on self-reported physical activity at baseline are included in this paper and any change in this behaviour during follow-up, or indeed random or systematic measurement error, may have led to misclassification and attenuated any observed associations.

Weight loss may be classified as intentional or unintentional. Participants may make conscious efforts to lose weight, through changes in diet and/or exercise. A recent meta-analysis of 15 randomised controlled trials in obese older adults found that intentional weight loss may be associated with approximately a 15% reduction in all-cause mortality [50], whilst others, in agreement with our findings, observed a higher mortality risk [51, 52]. Alternatively, weight loss may be due to illness or the diagnosis of a chronic disease. Recent data from the Longitudinal Aging Study Amsterdam show that unintentional weight loss in the past 6 months due to medical or unknown reasons or due to a change in eating pattern (unintentional or intentional) was associated with an increased 3-year mortality risk among community-dwelling men and women, aged ≥ 55 years [53]; this finding relating to unintentional weight loss is in agreement with previous studies [15, 54], including data from this study. A study of 4331 older men concluded that those who lost (− 5%) weight, total lean mass, or total fat mass over a 4.6 years period had a higher risk of mortality than those whose weight remained stable [17].

We also explored the association of weight change and all-cause mortality after excluding those who said that they had lost more than 5 kg (10 lbs) in the last 5 years, due to illness and in a subgroup who said that they had lost weight due to dieting. Once again, we found that the association of a higher HR with weight loss was still evident. However, numerous studies over the last 20–30 years have suggested that adults who diet in order to lose weight are more likely to gain weight in the future and even become obese [55–57]. In this analysis, of those participants who said that they had lost more than 5 kg due to dieting during the 5 years before the 2HE, 29% of them had actually gained weight between health examinations. It is possible that during this time period, they did lose weight but then regained it, and possibly more, but we are unable to verify this. Additionally, recent reviews have shown that normal-weight individuals who diet to lose weight are more likely to gain weight in the future than non-dieters [58, 59] and that dieting in those of normal weight compared to those who are overweight or obese may be a stronger predictor of future weight gain [56]. Zheng et al. [60] found that of six BMI trajectories, those who were in the overweight stable trajectory had the lowest mortality risk whereas those of normal weight who lost weight had the second highest mortality risk in a study of 9538 adults aged 51–77 years from the US Health and Retirement Study. Results from a prospective, population-based cohort study of 1975 men and women, aged 70–79 years, found that, over a 5 years period, weight cycling was associated with higher mortality risk in women: HR 1.62 (CI 1.15–2.30) and in men: HR 1.50 (CI 1.08–2.08) [18]. Weight cycling was also found to be a risk factor for mortality in the Cardiovascular Health Study [16], after adjustment for demographic risk factors, height, self-reported health and comorbidities: HR 1.66 (CI 1.38–2.00). We expressed the mean absolute annual weight change for those who said that they had lost weight due to dieting by BMI category at 1HE (Supplementary Table 5). Women who have a normal weight at baseline (BMI ≥ 20 and < 25 kg/m^2^) had a mean annual weight increase of 0.47 kg/year (SD 1.33); men who said that they lost weight by dieting did have a mean weight loss in each of the three BMI categories. These data on dieting in normal-weight women in our study provide further evidence of a subsequent gain in weight, and highlight the importance of objective weight measurements.

### Public health considerations

Weight or BMI do not simply reflect fat mass but also bone and lean body mass or muscle. Thus, weight loss may indicate not just fat loss but also loss in lean body mass, which may be particularly relevant in an ageing population, as weight loss and weight cycling in older adults are considered problematic because recovery of muscle mass is difficult [61–63]. Whereas, individuals who maintain body weight in later life may be those who are more likely to maintain bone mass and muscle compared to those who lose weight [40, 64]. Lee et al. [17] found that older men who lost (− 5%) weight, total lean mass, or total fat mass over a 4.6 years period had a higher risk of mortality than those who maintained their weight. Rapid weight loss and decreased muscle mass and strength are commonly associated with frailty, which is associated with mortality [65]. Some excess body weight in pre-frail and frail adults in later life may be beneficial, as may interventions to maintain or promote weight gain in frail older adults [66]. Weight management plans for obese, elderly individuals should therefore be specifically tailored in an effort to maintain or increase quality of life and physical function [67, 68]. A recent systematic review and meta-analysis found that weight-reducing diets for obese adults were associated with a 18% relative reduction in all-cause mortality but the authors also conclude that their findings support public health measures to prevent weight gain [69]. Yang et al. found an inverse relationship between lung cancer survival and weight loss at presentation and a potentially protective effect of obesity [36], in the form of greater physiological reserves, (excess fat and muscle), which may also be beneficial in other diseases displaying high catabolic states. The recent NICE guideline recommends that staff are trained to deliver multicomponent programmes that cover weight management, dietary habits, safe physical activity and behaviour-change strategies and that this should include the ability to adapt interventions to individual needs [8]. Given the wealth of evidence on the health consequences of obesity, efforts should perhaps be focussed on young adults [70] regarding the importance of lifestyle, including adequate nutrition and physical activity, and of achieving and maintaining a healthy weight in earlier adulthood.

## Conclusion

In summary, weight loss of more than 2.5 kg over an interval of approximately 4 years is associated with a higher mortality over 15 years of follow-up in this population-based cohort study of 12,580 middle-aged and elderly men and women. However, the potential presence of undiagnosed pre-existing disease and the inability to take weight cycling into account need to be remembered when interpreting these results. Unravelling the causal pathways underlying the observed association between objectively measured weight loss and subsequent higher mortality risk in this population-based study will require more detailed studies, including that of changes in body composition, such as muscle mass.

## Electronic supplementary material

Below is the link to the electronic supplementary material.

Supplementary material 1 (DOCX 38 kb)

## Abbreviations

EPIC: European Prospective Investigation into Cancer and Nutrition
BMI: Body mass index
CVD: Cardio-vascular disease
