# Supplementary material for: Weight change and 15 year mortality: results from the European Prospective Investigation into Cancer in Norfolk (EPIC-Norfolk) cohort study
Source: Eur J Epidemiol. 2017 Dec 20;33(1):37–53. doi: 10.1007/s10654-017-0343-y (PMC5803289; doi:10.1007/s10654-017-0343-y)
Supplement: Supplementary file 1 — Supplementary material 1 (DOCX 38 kb) [file 10654_2017_343_MOESM1_ESM.docx]

**Supplementary table 1** Baseline characteristics of EPIC-Norfolk men and women who attended 1HE, and those who attended both 1HE and 2HE, before and after exclusion criteria were applied. Continuous variables are Mean (SD) and categorical variables are n (%).

|  |  | **Men** |  |  |  | **Women** |  |
| --- | --- | --- | --- | --- | --- | --- | --- |
|  | 1HE  N=11607 | 1HE and 2HE  N=6582 | 1HE and 2HE with exclusions^a^  N=5479 |  | 1HE  N=14032 | 1HE and 2HE  N=8446 | 1HE and 2HEwith exclusions^a^  N=7101 |
| Weight, kg | 80.4 (11.5) | 80.1 (11.0) | 80.2 (10.9) |  | 67.9 (11.8) | 67.4 (11.3) | 67.5 (11.1) |
| Age, years | 59.6 (9.3) | 59.8 (8.9) | 59.0 (8.9) |  | 58.9 (9.3) | 58.4 (8.9) | 57.8 (8.8) |
| BMI, kg/m2 | 26.5 (3.3) | 26.4 (3.2) | 26.3 (3.1) |  | 26.6 (4.4) | 25.9 (4.1) | 25.9 (4.1) |
| Smoking status |  |  |  |  |  |  |  |
| Current | 1405 (12.2) | 616 (9.4) | 509 (9.3) |  | 1579 (11.4) | 764 (9.1) | 641 (9.0) |
| Former | 6284 (54.5) | 3563 (54.5) | 2902 (53.0) |  | 4477 (32.2) | 2632 (31.4) | 2239 (31.5) |
| Never | 3837 (33.3) | 2355 (36.0) | 2068 (37.7) |  | 7837 (56.4) | 4977 (59.4) | 4221 (59.4) |
| Physical activity |  |  |  |  |  |  |  |
| Inactive | 3586 (30.9) | 1826 (27.7) | 1446 (26.4) |  | 4277 (30.5) | 2226 (26.4) | 1761 (24.8) |
| Moderately inactive | 2858 (24.6) | 1649 (25.1) | 1356 (24.8) |  | 4493 (32.0) | 2760 (32.7) | 2360 (33.2) |
| Moderately active | 2660 (22.9) | 1632 (24.8) | 1381 (25.2) |  | 3116 (22.2) | 2018 (23.9) | 1722 (24.2) |
| active | 2502 (21.6) | 1475 (22.4) | 1296 (23.6) |  | 2146 (15.3) | 1442 (17.1) | 1258 (17.7) |
| Social class |  |  |  |  |  |  |  |
| Non-manual | 6657 (58.4) | 4034 (62.2) | 3401 (62.1) |  | 8394 (61.4) | 5304 (64.0) | 4502 (63.4) |
| Manual | 4744 (41.6) | 2451 (37.8) | 2078 (37.9) |  | 5274 (38.6) | 2978 (36.0) | 2599 (36.6) |
| Educational level |  |  |  |  |  |  |  |
| No qualifications | 3534 (30.5) | 1801 (27.4) | 1447 (26.4) |  | 5920 (42.2) | 3169 (37.5) | 2598 (36.6) |
| O level and above | 8064 (69.5) | 4779 (72.6) | 4032 (73.6) |  | 8103 (57.8) | 5272 (62.5) | 4503 (63.4) |
| Self-reported diseases |  |  |  |  |  |  |  |
| CVD (yes) | 798 (6.9) | 394 (6.0) |  |  | 324 (2.3) | 160 (1.9) |  |
| Cancer (yes) | 450 (3.9) | 233 (3.5) |  |  | 960 (6.8) | 568 (6.7) |  |
| Asthma (yes) | 892 (7.7) | 485 (7.4) |  |  | 1271 (9.1) | 752 (8.9) |  |
| Bronchitis (yes) | 1029 (8.9) | 560 (8.5) |  |  | 1337 (9.5) | 788 (9.3) |  |
| Deaths | 3995 (34.4) | 2028 (30.8) | 1421 (25.9) |  | 3432 (24.5) | 1627 (19.3) | 1182 (16.6) |
| Weight change categories |  |  |  |  |  |  |  |
| loss >5 kg |  | 275 (4.2) | 215 (3.9) |  |  | 454 (5.4) | 362 (5.1) |
| loss >2.5 & ≤5 kg |  | 505 (7.7) | 423 (7.7) |  |  | 634 (7.5) | 517 (7.3) |
| Reference |  | 3565 (54.2) | 2983 (54.4) |  |  | 4377 (51.8) | 3690 (52.0) |
| gain >2.5 & ≤5 kg |  | 1420 (21.6) | 1206 (22.0) |  |  | 1802 (21.3) | 1540 (21.7) |
| gain >5 & ≤10 kg |  | 713 (10.8) | 577 (10.5) |  |  | 979 (11.6) | 841 (11.8) |
| gain >10 kg |  | 104 (1.6) | 75 (1.4) |  |  | 200 (2.4) | 151 (2.1) |

^a^ self-reported cancer and CVD, missing data on weight, height, smoking status, social class, educational level and BMI < 18.5 kg/m^2^

*BMI* body mass index, *1HE* 1st health examination, *2HE* 2nd health examination

**Supplementary table 2**. Association between weight change and all-cause mortality, adjusted for all considered variables.

|  | | HR | | P>z | | 95% CI | | |
| --- | --- | --- | --- | --- | --- | --- | --- | --- |
| **MEN (1421/5479)** |  | |  | |  | |  |  |
| Weight change category |  | |  | |  | |  |  |
| loss >5 kg | 1.83 | | 0.001 | | 1.46 | | 2.29 |  |
| loss >2.5 & ≤5 kg | 1.29 | | 0.001 | | 1.09 | | 1.54 |  |
| loss or gain ≤ 2.5 kg | Ref | |  | |  | |  |  |
| gain >2.5 & ≤5 kg | 0.94 | | 0.382 | | 0.81 | | 1.08 |  |
| gain >5 & ≤10 kg | 1.01 | | 0.893 | | 0.84 | | 1.23 |  |
| gain >10 kg | 1.49 | | 0.080 | | 0.95 | | 2.33 |  |
|  |  | |  | |  | |  |  |
| Age, (1HE), per year | 1.13 | | 0.001 | | 1.12 | | 1.14 |  |
|  |  | |  | |  | |  |  |
| Smoking status (1HE) |  | |  | |  | |  |  |
| Never | Ref | |  | |  | |  |  |
| Current | 2.55 | | 0.001 | | 2.13 | | 3.06 |  |
| Former | 1.40 | | 0.001 | | 1.24 | | 1.59 |  |
|  |  | |  | |  | |  |  |
| BMI (1HE), per kg/m^2^ | 1.01 | | 0.159 | | 1.00 | | 1.03 |  |
|  |  | |  | |  | |  |  |
| Physical activity (1HE) |  | |  | |  | |  |  |
| Inactive | Ref | |  | |  | |  |  |
| Moderately inactive | 0.87 | | 0.044 | | 0.76 | | 1.00 |  |
| Moderately active | 0.80 | | 0.003 | | 0.70 | | 0.93 |  |
| Active | 0.86 | | 0.046 | | 0.74 | | 1.00 |  |
|  |  | |  | |  | |  |  |
| Social class (1HE) |  | |  | |  | |  |  |
| Non-manual | Ref | |  | |  | |  |  |
| Manual | 1.08 | | 0.164 | | 0.97 | | 1.22 |  |
|  |  | |  | |  | |  |  |
| Educational level (1HE) |  | |  | |  | |  |  |
| No qualifications | Ref | |  | |  | |  |  |
| O level & above | 0.98 | | 0.800 | | 0.88 | | 1.11 |  |
|  |  | |  | |  | |  |  |
| **WOMEN (1182/7101)** |  | |  | |  | |  |  |
| Weight change category |  | |  | |  | |  |  |
| loss >5 kg | 1.68 | | 0.001 | | 1.34 | | 2.10 |  |
| loss >2.5 & ≤5 kg | 1.32 | | 0.005 | | 1.09 | | 1.60 |  |
| loss or gain ≤ 2.5 kg | Ref | |  | |  | |  |  |
| gain >2.5 & ≤5 kg | 0.93 | | 0.375 | | 0.80 | | 1.09 |  |
| gain >5 & ≤10 kg | 1.11 | | 0.328 | | 0.90 | | 1.36 |  |
| gain >10 kg | 0.98 | | 0.931 | | 0.58 | | 1.64 |  |
|  |  | |  | |  | |  |  |
| Age, (1HE), per year | 1.13 | | 0.001 | | 1.12 | | 1.14 |  |
|  |  | |  | |  | |  |  |
| Smoking status (1HE) |  | |  | |  | |  |  |
| Never | Ref | |  | |  | |  |  |
| Current | 2.13 | | 0.001 | | 1.76 | | 2.58 |  |
| Former | 1.16 | | 0.022 | | 1.02 | | 1.31 |  |
|  |  | |  | |  | |  |  |
| BMI (1HE), per kg/m^2^ | 1.00 | | 0.488 | | 0.99 | | 1.02 |  |
|  |  | |  | |  | |  |  |
| Physical activity (1HE) |  | |  | |  | |  |  |
| Inactive | Ref | |  | |  | |  |  |
| Moderately inactive | 0.82 | | 0.006 | | 0.72 | | 0.95 |  |
| Moderately active | 0.80 | | 0.007 | | 0.68 | | 0.94 |  |
| Active | 0.74 | | 0.003 | | 0.60 | | 0.90 |  |
|  |  | |  | |  | |  |  |
| Social class (1HE) |  | |  | |  | |  |  |
| Non-manual | Ref | |  | |  | |  |  |
| Manual | 0.98 | | 0.779 | | 0.87 | | 1.11 |  |
|  |  | |  | |  | |  |  |
| Educational level (1HE) |  | |  | |  | |  |  |
| No qualifications | Ref | |  | |  | |  |  |
| O level & above | 0.82 | | 0.001 | | 0.72 | | 0.92 |  |

adjusted for 1HE values of age (continuous), smoking status (categorical), BMI (continuous), physical activity (categorical), social class (categorical) and educational level (categorical)

*1HE* 1st health examination, *BMI* body mass index

**Supplementary table 3** Cox multivariable-adjusted HRs^a^ for the association between weight change category and all-cause mortality in 12580 men and women, stratified by smoking history.

|  |  |  |  |  |  | **Weight change categories** | | |  | |  |
| --- | --- | --- | --- | --- | --- | --- | --- | --- | --- | --- | --- |
|  |  |  |  | loss >5 kg  (n=577) | loss >2.5 & ≤5 kg  (n=940) | loss or gain ≤2.5 kg  (n=6673) | gain >2.5 & ≤5 kg  (n=2746) | gain >5 & ≤10 kg  (n=1418) | | gain >10 kg  (n=226) | |
|  |  |  |  |  |  |  |  |  | |  | |
|  | Weight change, mean (SD), kg | N | Events | HR (95% CI) | HR (95% CI) |  | HR (95% CI) | HR (95% CI) | | HR (95% CI) | |
|  |  |  |  |  |  |  |  |  | |  | |
| Longterm smokers (current-current) | 1.1 (4.0) | 897 | 253 | * 1.58 (1.01 - 2.46) | 1.18 (0.83 - 1.70) | Ref | ** 0.61 (0.42 - 0.88) | 0.87 (0.53 - 1.43) | | 0.82 (0.26 - 2.63) | |
|  |  |  |  |  |  |  |  |  | |  | |
| Recent smokers (never/former - current) | 0.2 (3.6) | 112 | 25 | 0.63 (0.08 - 5.10) | 2.41 (0.60 – 9.64) | Ref | 1.21 (0.31 – 4.70) | 1.34 (0.29 - 6.24) | | ^b^ | |
|  |  |  |  |  |  |  |  |  | |  | |
| Longterm stoppers (former- former) | 1.4 (3.9) | 5038 | 1268 | *** 1.57 (1.23 - 2.02) | *** 1.40 (1.17 - 1.69) | Ref | 0.95 (0.82 - 1.11) | 1.06 (0.87 - 1.30) | | 1.25 (0.79 - 1.97) | |
|  |  |  |  |  |  |  |  |  | |  | |
| Recent stoppers  (current - former) | 3.4 (4.8) | 253 | 65 | 1.71 (0.47 – 6.24) | 2.29 (0.74 – 7.09) | Ref | 1.34 (0.69 - 2.60) | 1.02 (0.52 – 2.03) | | 1.21 (0.27 – 5.48) | |
|  |  |  |  |  |  |  |  |  | |  | |
| Never smokers  (never - never) | 1.3 (3.8) | 6280 | 992 | *** 1.98 (1.56 - 2.53) | 1.19 (0.95 - 1.48) | Ref | 0.98 (0.83 - 1.16) | 1.14 (0.90 - 1.44) | | 1.43 (0.78 - 2.62) | |

^a^Adjusted for age, sex, BMI, physical activity, social class and educational level

^b^Zero participants in category

Significance of HRs: ****P*<0.001; ** *P*<0.01; * *P*<0.05

*HR* hazard ratio, *CI* confidence interval

**Supplementary table 4** Association between weight change and all-cause mortality in 5479 men and 7101 women, assessed using Cox proportional hazards regression with a median follow-up from 2HE of 15 years. Results are hazard ratios and 95% confidence intervals, HR (95%CI).

|  |  |  | **Weight change categories** | |  | |  | |
| --- | --- | --- | --- | --- | --- | --- | --- | --- |
|  | **loss >5 kg** | **loss >2.5 & ≤5 kg** | **loss or gain ≤2.5 kg** | **gain >2.5 & ≤5 kg** | **gain >5 & ≤10 kg** | | **gain >10 kg** | |
|  |  |  |  |  |  | |  | |
| **MEN, N** | 215 | 423 | 2983 | 1206 | 577 | | 75 | |
| ALL CAUSE MORTALITY |  |  |  |  |  | |  | |
| Number of events (%) | 91 (41.9) | 154 (36.2) | 801 (26.5) | 259 (21.1) | 128 (21.9) | | 20 (26.3) | |
| Model 2 | *** 1.92 (1.54 - 2.40) | ** 1.31 (1.10 - 1.56) | Ref | 0.94 (0.81 - 1.08) | 1.03 (0.85 - 1.25) | | 1.51 (0.97 - 2.36) | |
| Model 3 | *** 1.84 (1.47 - 2.30) | ** 1.30 (1.09 - 1.54) | Ref | 0.94 (0.82 - 1.09) | 1.02 (0.84 - 1.24) | | 1.50 (0.96 - 2.34) | |
|  |  |  |  |  |  | |  | |
| **WOMEN, N (%)** | 362 | 517 | 3690 | 1540 | 841 | 151 | |  |
| ALL CAUSE MORTALITY |  |  |  |  |  |  | |  |
| Number of events (%) | 94 (25.4) | 128 (24.3) | 651 (17.3) | 223 (14.2) | 115 (13.5) | 15 (9.8) | |  |
| Model 2 | *** 1.71 (1.37 - 2.13) | ** 1.31 (1.08 - 1.58) | Ref | 0.94 (0.81 - 1.10) | 1.14 (0.93 - 1.39) | 1.05 (0.62 - 1.75) | |  |
| Model 3 | *** 1.65 (1.32 – 2.07) | ** 1.31 (1.08 - 1.58) | Ref | 0.93 (0.80 - 1.09) | 1.12 (0.91 - 1.37) | 1.00 (0.60 - 1.67) | |  |

Model 2: adjusted for age (continuous) and smoking history (categorical, as defined in Supplementary table 3)

Model 3: Model 2 and further adjusted for BMI (continuous), physical activity (categorical), social class (categorical) and educational level (categorical)

Significance of HRs: ****P*<0.001; ** *P*<0.01; * *P*<0.05

**Supplementary table 5** Mean annual weight changes in 1044 men and women who stated that they lost more than 5 kg in the last 5 years (HLQ2), due to dieting, by BMI category at 1HE.

|  |  | **Men** |  |  |  | **Women** |  |
| --- | --- | --- | --- | --- | --- | --- | --- |
|  |  | Weight change (kg/y) | |  |  | Weight change (kg/y) | |
|  | N | Mean | SD |  | N | Mean | SD |
| By BMI (1HE) |  |  |  |  |  |  |  |
| ≥ 18.5 & < 25 kg/m^2^ | 25 | -0.32 | 1.06 |  | 164 | 0.47 | 1.33 |
|  |  |  |  |  |  |  |  |
| ≥ 25 & < 30 kg/m^2^ | 154 | -0.21 | 1.47 |  | 380 | 0.06 | 1.65 |
| ≥ 30 kg/m^2^ | 88 | -0.10 | 1.83 |  | 233 | -0.44 | 2.17 |
|  |  |  |  |  |  |  |  |

*HLQ2* 2nd Health and Lifestyle Questionnaire, *BMI* body mass index, *1HE* 1st health examination, *SD* standard deviation
